# Concentración de cortisol salival con un enfoque de género en las diferentes etapas del desarrollo humano. Una revisión de la literatura

**DOI:** 10.21142/2523-2754-0903-2021-074

**Published:** 2021-10-06

**Authors:** Isaac Francisco Gómez, Virginia Cecilia Rosende, Rolando Pablo Alejandro Juárez

**Affiliations:** 1 Grupo de Investigación y Desarrollo: Saliva como Fluido Diagnóstico, Facultad de Odontología, Universidad Nacional del Nordeste. Corrientes, Argentina. ifg9518@gmail.com, vcrosende@odn.unne.edu.ar, ropablojuarez@odn.unne.edu.ar Universidad Nacional del Nordeste Grupo de Investigación y Desarrollo: Saliva como Fluido Diagnóstico Facultad de Odontología Universidad Nacional del Nordeste Corrientes Argentina ifg9518@gmail.com vcrosende@odn.unne.edu.ar ropablojuarez@odn.unne.edu.ar

**Keywords:** cortisol, saliva, género, grupos de edad, cortisol, saliva, gender, age groups

## Abstract

La secreción del cortisol salival (Corts) es un reflejo de la adaptación de los individuos a los estresores internos/externos, con varios factores involucrados: biológicos y sociales. El objetivo de este estudio fue realizar una investigación documental sobre las variaciones de la concentración del Corts en relación con las diferencias biológicas de sexo y socialización de género, en las diferentes etapas del desarrollo humano. Para la búsqueda de la evidencia científica se utilizaron Medline/PubMed y Google Académico. Se seleccionaron 20 artículos originales, en idioma inglés, publicados en el periodo 2010-2020, incluidos ensayos clínicos y estudios observacionales. La relación entre Corts y género se discutió históricamente considerando los aspectos evolutivos y biológicos; sin embargo, no puede explicarse como una visión causa-efecto. Depende de factores socioculturales únicos que, en periodos sensibles del desarrollo humano, alteran el eje hipotalámico-pituitario-adrenal y condicionan el afrontamiento del estrés.

## Abstract (en)

Salivary cortisol secretion (Corts) is an individual adaptation reflex to internal / external stressors, which involves several biological and social factors. The aim of this study was to perform a review on variations in Corts concentrations in relation to biological differences of sex and gender socialization in the different stages of human development. MEDLINE / PubMed and Google Scholar were used to search for scientific evidence. 20 original English-language articles published from 2010-2020 were selected, including clinical trials and observational studies. The relationship between Corts and gender was historically discussed considering evolutionary and biological aspects. However, it cannot be explained as a cause-effect view and depends on unique sociocultural factors, which in sensitive periods of human development alter the hypothalamic-pituitary-adrenal axis and condition coping with stress.

## INTRODUCCIÓN

El cortisol (Cort) o hidrocortisona es una hormona esteroidea o glucocorticoide, secretada en la zona fascicular de la corteza de la glándula suprarrenal. Es el principal glucocorticoide adrenal, con receptores en casi todos los tejidos del cuerpo, y cumple varias funciones metabólicas, como inducir la movilización de energía; aumentar las tasas de perfusión cerebral y la utilización local de glucosa; mejorar la producción cardiovascular y respiratoria, y la redistribución del flujo sanguíneo; aumentar la entrega de sustrato y energía al cerebro y los músculos; y modular la función inmunitaria ^1^. Su secreción está regulada por el eje hipotalámico-pituitario-adrenal (HPA), a través de la secreción de la hormona liberadora de corticotropina (CRH) y la adenocorticotropina (ACTH). En respuesta a diversos procesos biológicos y psicológicos, la secreción de CRH por parte del hipotálamo desencadena la liberación en la hipófisis de ACTH, lo que produce un incremento en la secreción de Cort en la corteza suprarrenal. Además de la CRH, la vasopresina se secreta conjuntamente en la sangre portal y estimula, sinérgicamente con la CRH, la secreción de ACTH a nivel de la pituitaria anterior ^2^.

El Cort exhibe una gran variación diurna, gobernada por el ritmo circadiano de la ACTH ^3^. Las concentraciones de Cort salival aumentan por la mañana y alcanzan su punto máximo aproximadamente 30 minutos después del despertar, para ir disminuyendo gradualmente a lo largo del día, hasta llegar a un mínimo durante la primera y segunda horas del sueño (primera mitad de la noche). Luego, sus niveles suben en forma gradual en las fases ulteriores del sueño (segunda mitad de la noche) para volver a un máximo al despertar ^4^^,^^5^. Sin embargo, si se altera el patrón del sueño, el ritmo circadiano de la ACTH puede modificarse. Por ejemplo, en el caso de las personas con privación aguda parcial o total del sueño, los niveles de Cort se alteran. Asimismo, los ciclos de luz y oscuridad también influyen sobre el ritmo circadiano y sobre la secreción de Cort ^6^. Además del ciclo diurno, existe un rápido aumento de los niveles de Cort por la mañana, entre los 20 y 30 minutos después del despertar, denominado respuesta de despertar del Cort (CAR). Surge cuando el organismo se enfrenta a una serie de demandas futuras, como la carga laboral o los torneos competitivos ^3^^,^^4^. 

El eje HPA es el componente central de los sistemas de respuesta al estrés y las concentraciones de Cort se utilizan como un indicador de estrés biológico ^7^. Su determinación bioquímica puede realizarse a través de una muestra de sangre, orina o saliva ^8^. En adultos jóvenes, el valor de referencia de los niveles séricos es de 19,28 ± 3,56 µg/100 ml ^9^; por la mañana, de 5 a 25 µg/dL; y al final del día, menores a 10 mcg/dL ^5^. Además de estas variaciones diurnas, se observaron amplias variaciones inter e intraindividuales, que pueden explicarse en parte por la sincronización de la muestra en relación con un pulso de liberación de Cort ^10^. 

En plasma normal, el Cort libre representa menos del 6% del total, con un 80-90% unido a la globulina transportadora de corticosteroides (CBG) y un 5 al 10% a la albúmina ^11^. El método de laboratorio para medir el Cort sérico libre no es factible para el uso rutinario. Como sustituto, se utiliza la prueba del Cort salival, que solo necesita un pequeño volumen de saliva, es fácil de efectuar, se puede realizar a diario, su técnica no es invasiva, refleja el nivel de Cort libre biológicamente activo en suero, no se ve afectada por variaciones en el CBG y es independiente del flujo salival ^12^^-^^14^. 

El Cort salival ha sido reconocido durante mucho tiempo como un marcador de la actividad del eje HPA ^15^ y hay varias publicaciones sobre qué tener en cuenta al recolectar saliva para la evaluación del Cort salival ^16^. Su concentración en saliva no estimulada matutina (de 9 a 9:15 a. m.) es de 5,34 ± 1,33 ng/ml, medido con ELISA (EIA, Diametra kit, Corea), con valores guía, de acuerdo con el kit, en el rango de 3 a 10 ng/ml por la mañana y de 0,6 a 2,5 ng/ml por la noche ^17^. Hoy en día, el Cort salival se utiliza de forma rutinaria como biomarcador de estrés psicológico y enfermedades mentales o físicas relacionadas ^2^. En la clínica odontológica, se ha utilizado para evaluar el papel de estrés en relación con caries dental ^18^, enfermedad periodontal ^19^, hábitos parafuncionales ^20^ y procedimientos odontológicos ^21^. En estos casos, se observaron diferencias de respuesta entre individuos, ocasionados por varios factores internos y externos modificadores de la respuesta del eje HPA. Estas variables adicionales, como la sensibilidad suprarrenal y la carga de estrés, afectan los niveles de Cort total y libre en sangre, y finalmente los niveles de Cort en saliva ^22^. Otros factores determinantes que deben ser tenidos en cuenta para una medición confiable del Cort salival, para modular el ritmo circadiano del Cort y el CAR son edad, género, actividad física, calidad del sueño, dieta e ingesta de fármacos ^23^^,^^24^. 

Los estudios sobre ritmos circadianos de secreción de cortisol y el CAR, han mostrado resultados diferentes en relación con la edad y las características sexuales. Esto puede deberse a las características de la muestra y las técnicas de análisis de los diferentes estudios ^3^. Los esteroides sexuales son moduladores importante del HPA, con una actividad bastante compleja y solo parcialmente comprendida. Por ejemplo, las mujeres muestran niveles de Cort y CAR en saliva diferentes que los hombres, según la fase del ciclo menstrual, el uso de anticonceptivos orales, la menopausia y por influencia de los ritmos circadianos ^2^^,^^22^. Asimismo, aunque las mujeres mostraron un aumento de CAR salival prácticamente idéntico en comparación con los hombres, se observó luego una disminución retardada. En todos los casos, las diferencias fueron del 1 al 4% (aproximadamente 3 nmol/l) a los 60 minutos del despertar, y no se pudo determinar si tenían efectos funcionales sobre los tejidos diana ^3^^,^^5^.

Para realizar un estudio con enfoque de género, es necesario comprender dos conceptos fundamentales: diferencias biológicas de sexo y género sociocultural. El sexo biológico constituye una serie de factores de sesgo sexual que incluyen diferencias en genes, anatomía, gónadas y hormonas, mientras que la socialización de género se refiere a un espectro de disimilitudes implícitas y explícitas entre hombres y mujeres en roles, identidades y orientaciones socialmente construidos ^25^^-^^27^.

El objetivo de esta revisión narrativa fue la recopilación y análisis de información científica sobre las variaciones de la concentración del Cort en saliva, en relación con las diferencias biológicas de sexo y de socialización de género, en las diferentes etapas del desarrollo humano. 

## METODOLOGÍA

Para realizar la búsqueda de la evidencia científica, se utilizaron las siguientes fuentes de información: Medline/PubMed y Google Académico. Se utilizaron como términos de búsqueda las palabras clave: “cortisol”, “saliva”, “gender”, relacionadas entre sí con el término booleano AND. 

**Figura. 1:**
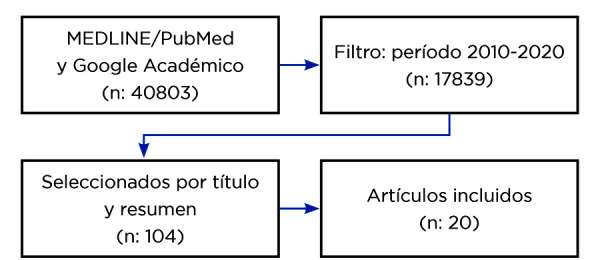
Diagrama de flujo para la selección de artículos

Los criterios de inclusión fueron artículos en idioma inglés, con un límite de 10 años de antigüedad (2010-2020), incluidos ensayos clínicos y estudios observacionales. Se seleccionaron 20 artículos originales (Figura 1, Tabla 1), según el nivel de evidencia y el grado de recomendación de las pautas del Centro de Medicina Basada en la Evidencia, de la Universidad de Oxford ^28^.

**Tabla I:** Artículos incluidos en la revisión

Autor	Año	Cort/edad y género
DiPietro *et al*.	2011	Embarazadas durante la última mitad de la gestación. Cort materno libre en relación con el sexo fetal.
Hatzinger *et al*.	2012	Edad preescolar. SF con mayor secreción.
Bosch *et al*.	2012	Adolescentes. Relación con la edad. Los efectos no fueron modificados por sexo.
Edelman *et al*.	2012	Edad promedio: 25 años. Mayor secreción en SM.
Walker *et al*.	2013	Individuos de 12 a 30 años. Efecto de la edad, pero no del sexo.
Hatzinger *et al*.	2013	Niños de edad preescolar. Cort en SF.
Sjörs *et al*.	2014	Edades 25 a 50 años. El estrés no laboral mostró asociación con las mujeres ( Cort).
Kogler *et al*.	2015	Edad: 24 años promedio. No se observaron diferencias de sexo en Cort.
Jacobson *et al*.	2015	Hombres heterosexuales y homosexuales con edades medias entre 24 y 26 años. No hubo diferencias de Cort.
Juster *et al*.	2015	Adultos (edad media, 25 años). En heterosexuales Cort en SM. Mujeres lesbianas/ bisexuales Cort que mujeres heterosexuales.
Moskow *et al*.	2016	Adolescentes. Cort en relación con la edad, pero no con el sexo.
Juster *et al*.	2016	Adultos (edad media, 25 años). Cort en SF heterosexuales asociado con testosterona y progesterona. Cort en SM homosexuales/ bisexuales asociado con testosterona.
Andiarena *et al*.	2017	14 meses-4años. Interacciones significativas entre el sexo y Cort.
Coelli *et al*.	2017	Edades 18-74. Cort en relación con edad, pero no con género.
Helbig & Backhaus	2017	Edad promedio: 24 años. No hubo diferencias de sexo en la liberación de cortisol.
DuBois *et al*.	2017	Edad promedio: 32 años. Cort en hombres transgénero.
Samuel *et a*l.	2018	Adultos de 56-78 años. En concentración efecto de la edad, pero no de sexo. SF una más rápida en comparación con SM.
Manigault *et al*.	2018	Adultos jóvenes (18 a 35). Cort varía de acuerdo al contexto de revelación de la identidad sexual.
Sroykham & Wongsawat	2019	Edad promedio: 68-69 años. Cort levemente en hombres.
Stern *et al*.	2020	Edad promedio: 22 años. Relación de Cort con deseo sexual.

Cort: cortisol, : aumento, : disminución, SF: sexo femenino, SM: sexo masculino

La formulación final de la pregunta de investigación documental quedó redactada de la siguiente manera: ¿Cuáles son los efectos de las diferencias biológicas de sexo, en comparación con la socialización de género, sobre la concentración de cortisol salival en las diferentes etapas del desarrollo humano?

## DIFERENCIAS BIOLÓGICAS DE SEXO

### Programación del eje HPA

Existen etapas particulares (períodos sensibles) durante el desarrollo humano, en los que diversos factores influyen en la programación a largo plazo del eje HPA y la sensibilización a los factores estresantes ^29^. Asimismo, las diferencias individuales de la concentración del Cort en saliva están relacionadas con el desarrollo neuropsicológico a largo plazo, en el que el sexo biológico juega un papel importante ^30^.

### Embarazadas

El Cort salival materno difiere, según el sexo fetal, durante la segunda mitad del embarazo. Las mujeres gestantes con fetos masculinos presentan mayores niveles de Cort en saliva entre las 24 y 30 semanas de gestación, mientras que, a partir de las 30 semanas, se observó un mayor Cort salival materno en gestantes con fetos femeninos, nivel que persistió hasta el término del embarazo. Estos hallazgos pueden ser evidencia de retraso en la maduración, que refleja las adaptaciones específicas del sexo en una amplia gama de procesos fisiológicos ^31^. Por otra parte, la exposición perinatal y posnatal al estrés materno (estrés psicosocial materno prenatal, consumo de alcohol, bajo peso al nacer) puede afectar el eje HPA de los bebés y se han asociado constantemente con la hiperreactividad de HPA al estrés, un proceso conocido como programación prenatal. Incluso adultos con un HPA alterado, en comparación con los controles sanos, informaron retrospectivamente la exposición de las madres a eventos dramáticos de la vida y mostraron un patrón alterado en la secreción de Cort. Existen dos explicaciones probables: 1) las estructuras cerebrales involucradas en la regulación del eje HPA (hipocampo, corteza frontal y la amígdala), comienzan a desarrollarse prenatalmente y son extremadamente sensibles al estrés durante el período pre/posnatal; 2) las adversidades pre y posnatales, ocasionan una sensibilización al estrés y pueden aumentar la probabilidad de alteraciones del eje HPA inducidas por el estrés, después de la exposición a estresantes en edades posteriores ^29^^,^^32^.

### Infantes

A los 14 meses de edad, el Cort salival como marcador de estrés mostró una asociación con varios aspectos del desarrollo neuropsicológico (memoria, proceso motor y socioemocional), que se percibieron a los 4 años y un patrón de asociación específico por sexo, que podría deberse al hecho de que el eje HPA es, como la anatomía, la química y la función del cerebro, específico del sexo. Una posible explicación para estos efectos sexualmente dimórficos del Cort se puede encontrar en la literatura animal, que ha descrito importantes diferencias sexuales tanto en cómo los factores estresantes de la vida temprana afectan el desarrollo de la corteza prefrontal como en la conectividad de la misma con otras regiones del cerebro involucradas con la función cognitiva y la regulación emocional/conductual ^30^.

### Niños

En niños preescolares, se ha demostrado que el efecto del sexo biológico sobre la forma en que la actividad del eje HPA impacta en el funcionamiento psicológico. Así, en comparación con los niños, las niñas tienen una mayor secreción de Cort tanto a los 5 como a los 6 años. Además, los niños con niveles más altos de Cort tienen un mayor riesgo de desarrollar más dificultades psicológicas ^32^^,^^33^. 

Asimismo, en niños prepúberes, se observaron diferencias en la secreción de Cort con respecto al sexo que parecen ser independientes de los esteroides sexuales, más bien están asociadas con procesos psicosociales. Así, el marco de tiempo cuando ocurrió el evento de vida adverso modificó las secreciones futuras de Cort: si los eventos de vida adversos ocurrieron durante las edades de 6 a 11 años, los niveles de Cort aumentaron permanentemente; si se produjeron después de los 12 a 13 años, los niveles de Cort se redujeron de forma bastante permanente ^29^.

### Adolescentes y adultos

Durante la adolescencia, existe evidencia de que los procesos neuromaduracionales normales aumentan el estrés y los niveles de Cort ^34^. Los adolescentes son particularmente sensibles a las experiencias estresantes, cada estructura nerviosa implicada en la regulación del eje HPA tiene diferentes vías de maduración y, como resultado, períodos específicos para la exposición al estrés ^35^. 

Del mismo modo, se pudo determinar un aumento del Cort salival en relación con la edad en sujetos sanos entre 13 y 30 años ^35^. Las diferencias biológicas y psicológicas que aumentan después de la pubertad amplían la tendencia de las mujeres a experimentar y describir eventos como estresantes ^34^. Por ejemplo, en mujeres de mediana edad, sometidas a tensión normal de vida familiar y con demandas combinadas del hogar y el trabajo, se observó un ritmo de cortisol diurno aplanado. Asimismo, los factores psicosociales en el hogar, con una presión en el tiempo, desencadenaron estrés crónico no laboral y adaptación del eje HPA mediante la regulación a la baja en las mujeres ^36^. 

En una población sana con un rango de edades entre 18 a 74 años, la evaluación del Cort en saliva nocturno, una de las pruebas más fiables para detectar el síndrome de Cushing endógeno, no mostró diferencias con relación al sexo biológico ni al índice de masa corporal, pero sí una asociación positiva significativa entre los niveles de Cort salival y la edad, ligada a un posible cambio en la actividad del eje HPA con una secreción elevada de Cort que podría contribuir a la pérdida de masa ósea relacionada con el envejecimiento ^37^. Asimismo, el deterioro cognitivo es un proceso normal del envejecimiento, asociado con niveles elevados de Cort. En una población de edad avanzada, la actividad cerebral, la emoción y el Cort fueron influenciados por deterioros cognitivos, pero no se observaron diferencias significativas en la concentración del Cort en saliva entre sexos ^38^. Por otra parte, el Cort más bajo al despertar y su lenta disminución vespertina es un marcador del envejecimiento biológico del eje HPA de grupos vulnerables con una exposición diferencial a experiencias estresantes. Por ejemplo, en adultos afroamericanos mayores y de mediana edad con bajo nivel socioeconómico, no se observaron variaciones en el Cort al despertar según el sexo, aunque las mujeres presentaron una disminución más rápida ^39^.

### Respuestas fisiológicas y psicológicas al estrés

Con respecto a los mecanismos moleculares (neuroquímicos y neurogenéticos), se demostró que participan en las diferencias de sexo biológico de la reactividad del eje HPA. En este sentido, el epigenoma, con la modificación del receptor de glucocorticoides, contribuye a las disparidades de sexo. Además, en presencia de estradiol y serotonina, hay una mayor capacidad de respuesta del eje HPA a un factor de estrés social con aumentos en el Cort salival, especialmente en mujeres ^40^.

A su vez, el estrés afecta de manera diferencial a mujeres y hombres, y compromete claramente las regiones del cerebro involucradas en el control cognitivo y la modulación del procesamiento emocional. Así, los hombres parecen desactivar las regiones de procesamiento de emociones, mientras que las mujeres activan las áreas asociadas con los dominios motivacionales y emocionales. Sin embargo, la aplicación de una estrategia de regulación cognitiva conduce a una experiencia de estrés menos subjetiva en ambos sexos, sin diferencias significativas en la concentración del Cort en saliva. Sin embargo, en las mujeres se requiere más esfuerzo para regular cognitivamente el estrés, lo que probablemente desencadena un mayor estrés subjetivo que en los hombres ^41^.

Asimismo, las mujeres y los hombres difieren en su respuesta al estrés fisiológico y psicológico. En la evaluación del estrés cognitivo en un entorno naturalista, con control de la fase del ciclo menstrual y el uso de anticonceptivos orales para evitar resultados heterogéneos, no se observaron diferencias de sexo en el trazado de la liberación de Corts. Sin embargo, las mujeres mostraron una evaluación cognitiva desventajosa en comparación con los hombres y se sintieron subjetivamente más estresadas. En contraste, las respuestas fisiológicas al estrés fueron iguales entre mujeres y hombres. La falta de diferencia entre sujetos femeninos y masculinos con respecto a la reacción de estrés por Cort podría deberse a que el estrés psicológico y el nerviosismo no están fuertemente asociados con el Cort como el estrés fisiológico. Por otra parte, la alta respuesta subjetiva al estrés en las mujeres podría ser una reliquia de la evolución humana ^42^. 

## DIFERENCIAS POR SOCIALIZACIÓN DE GÉNERO

### La orientación sexual modula la reactividad del estrés endocrino

Las explicaciones biológicas del comportamiento homosexual, a menudo, han planteado que la desregulación de los perfiles hormonales específicos del género produce anomalías en el desarrollo neurológico de los circuitos relacionados con el comportamiento sexual específico de la especie. Sin embargo, se ha hipotetizado que tales diferencias de hormonas sexuales, que se supone son atribuibles a la orientación sexual, también están moduladas por fenómenos de estrés. En particular, el desarrollo de paradigmas de inducción de estrés basados en laboratorio ha demostrado que los sexos difieren en sus patrones de respuesta al estrés del Cort salival ^43^.

Así, se exploró si las minorías sexuales (edades entre 18 y 45 años) difieren del grupo control heterosexual del mismo sexo en términos de concentraciones salivales de testosterona, estradiol y progesterona antes y después de la exposición a la prueba de estrés social de Trier, en relación con la producción sistémica de Cort. Se observó que las hormonas sexuales varían según la orientación sexual en las mujeres, pero también de manera importante según los índices de estrés, y se confirmó que la orientación sexual modula la reactividad del Cort ^44^.

Por otra parte, la hipótesis de la hormona dual establece que el Cort y la testosterona interactúan y esta interacción podría ser un mejor predictor de las diferencias en los aspectos de la orientación sociosexual de los hombres que la testosterona sola. Las relaciones longitudinales y transversales entre la testosterona salival, el Cort, el deseo sexual informado y la sociosexualidad en una muestra de 61 hombres adultos jóvenes, demostraron que los efectos combinados de la testosterona y el Cort predicen el grado del interés de los hombres en el sexo casual. Sin embargo, no detectaron evidencia convincente de una asociación entre cambios hormonales dentro del sujeto y deseo sexual u orientación sociosexual ^45^.

### Atipicidad de género

Los comportamientos no conformes al género y la orientación sexual homosexual se han relacionado con niveles más altos de ansiedad. Al examinarse los efectos de la atipicidad de género y la orientación sexual en los niveles de ansiedad, después de una tarea de interacción social estresante, entre una muestra de hombres israelíes homosexuales con identidad revelada (edad promedio 27 años) y heterosexuales (edad promedio 25 años), los resultados mostraron que la atipicidad de género y la orientación sexual heterosexual predijeron niveles más altos de ansiedad por interacción social, pero no cambios en el Cort. Estos hallazgos sugieren que la atipicidad de género, no la homosexualidad, pone a las personas en riesgo de aumentar la ansiedad. En ese sentido, el estrés de las minorías puede estar más relacionado con el comportamiento de género individual que con la orientación sexual per se. La falta de cambios en el Cort salival, posiblemente, se deba a su falta de sensibilidad comparada con los autoinformes de los estados de ansiedad utilizados en la prueba de interacción social ^46^.

### Revelación de la identidad sexual 

El ocultamiento de la identidad sexual implica cierto grado de inhibición psicológica y carga cognitiva que resulta en malestar y angustia. A su vez, revelar la identidad sexual es un proceso complejo marcado por un cambio en los tipos de factores estresantes que enfrentan los adultos jóvenes de minorías sexuales. El grado de la divulgación influye en la función del eje HPA al actuar como un amortiguador de estrés, y el contexto de divulgación puede ser un moderador de esta relación (miembros de la familia, compañeros de trabajo, amigos o conocidos, y miembros de grupos religiosos). Así, la divulgación a los miembros de la familia produce una menor angustia psicológica y una reducción de la cantidad total de Cort producido en un día. Asimismo, la revelación temprana durante la adolescencia podría implicar resiliencia psicológica o relaciones familiares positivas, con una concentración de Cort diurno menor. Por lo tanto, en la medida en que la divulgación a los miembros de la familia limite la sobreexposición al cortisol, puede producir importantes efectos protectores del estrés en las poblaciones de minorías sexuales ^47^.

### Personas transgénero

Las personas transgénero, particularmente en contextos socioculturales donde las categorías de género masculino-femenino se aplican de manera rígida, experimentan un estrés significativo relacionado con la discriminación. La evaluación del funcionamiento del eje HPA, en relación con los factores estresantes específicos de la transición, basados en el estigma que experimentan los hombres transgénero durante su transición de mujer a hombre, mostraron una producción aumentada del Cort diurno. Los hombres transgénero sometidos a terapia con testosterona y que experimentaron estrés de identidad en transición tuvieron una lectura promedio de Cort al despertar de 1,43 puntos más alto que los hombres trans sin estrés de identidad. Después de estos niveles elevados, exhibieron una pendiente lineal más pronunciada y rápida. Los niveles más altos de Cort al despertar se presentaron con la gestión de una identidad social en transición y se relacionan con el riesgo percibido de humillación o falta de respeto, frente a situaciones como el uso de baños públicos específicos de género. En consecuencia, las diversas formas en las que los factores estresantes de las minorías, específicos de los desafíos que enfrentan los hombres trans durante su transición, pueden afectar las medidas del estrés biológico ^48^. 

## CONCLUSIONES

La relación entre Cort salival y características sexuales biológicas no puede explicarse como una visión causa-efecto. En realidad, la secreción de Cort salival es un reflejo de la adaptación de los individuos a los estresores internos/externos y depende de un contexto complejo, con varios factores involucrados que desencadenan una hiperactivación psicofisiológica y condicionan el afrontamiento del estrés. Los efectos de género en la capacidad de respuesta del eje HPA se discutieron históricamente considerando los aspectos evolutivos y biológicos. Sin embargo, el patrón de secreción del Cort salival presenta diferencias interindividuales complejas, que dependen de factores socioculturales únicos y el grado de vulnerabilidad de las personas.

Los efectos adversos o, por otro lado, las situaciones estresantes en periodos críticos del desarrollo humano, desde la exposición perinatal hasta la adultez, alteran el eje HPA, en función del sexo, la edad y los procesos psicosociales asociados. Las adversidades con efectos en la secreción del Cort salival que ocurrieron durante el periodo pre y posnatal, que se caracteriza por cambios cerebrales regionales subyacentes específicos, hacen a las personas más sensibles a los efectos de situaciones estresantes posteriores y persistentes sobre el eje HPA. Otro periodo crítico es la pubertad, dado que los adolescentes son particularmente sensibles a las experiencias estresantes y condicionan al eje HPA para las respuestas de Cort durante el resto de la vida. Los estudios futuros deben evaluar la secreción del Cort salival en relación con diferentes constructos psicosociales. 

## Fuente de financiamiento:

Secretaría General de Ciencia y Técnica. Programa de Becas de Investigación
